# Adjuvant role of macrophages in stem cell-induced cardiac repair in rats

**DOI:** 10.1038/s12276-018-0171-5

**Published:** 2018-11-05

**Authors:** Soo yeon Lim, Dong Im Cho, Hye-yun Jeong, Hye-jin Kang, Mi Ra Kim, Meeyoung Cho, Yong Sook Kim, Youngkeun Ahn

**Affiliations:** 1 Cell Regeneration Research Center, Chonnam National University Hospital, Gwangju, 61469 Republic of Korea; 2 Biomedical Research Institute, Chonnam National University Hospital, Gwangju, 61469 Republic of Korea; 3 Department of Cardiology, Chonnam National University Hospital, Gwangju, 61469 Republic of Korea

## Abstract

Bone marrow-derived mesenchymal stem cells (BMMSCs) are used extensively for cardiac repair and interact with immune cells in the damaged heart. Macrophages are known to be modulated by stem cells, and we hypothesized that priming macrophages with BMMSCs would enhance their therapeutic efficacy. Rat bone marrow-derived macrophages (BMDMs) were stimulated by lipopolysaccharide (LPS) with or without coculture with rat BMCs. In the LPS-stimulated BMDMs, induction of the inflammatory marker iNOS was attenuated, and the anti-inflammatory marker Arg1 was markedly upregulated by coculture with BMMSCs. Myocardial infarction (MI) was induced in rats. One group was injected with BMMSCs, and a second group was injected with MIX (a mixture of BMMSCs and BMDMs after coculture). The reduction in cardiac fibrosis was greater in the MIX group than in the BMC group. Cardiac function was improved in the BMMSC group and was substantially improved in the MIX group. Angiogenesis was better in the MIX group, and anti-inflammatory macrophages were more abundant in the MIX group than in the BMMSC group. In the BMMSCs, interferon regulatory factor 5 (IRF5) was exclusively induced by coculture with macrophages. IRF5 knockdown in BMMSCs failed to suppress inflammatory marker induction in the macrophages. In this study, we demonstrated the successful application of BMDMs primed with BMMSCs as an adjuvant to cell therapy for cardiac repair.

## Heart attacks: mixed cell therapy for heart regeneration

A tailored technique involving stem cells and anti-inflammatory immune cells shows promise for repairing heart tissue damage. Immune cells called anti-inflammatory macrophages are vital for healing of the heart following a heart attack. Youngkeun Ahn, Yong Sook Kim and co-workers at Chonnam National University Hospital in Gwangju, South Korea trialed a novel stem cell therapy on rats to improve cardiac repair. They took bone marrow-derived macrophages and stem cells from each rat and incubated the two cell types together to create individualized treatments. Following induced heart attacks, they injected one group of rats with both cell types, and another group with stem cells only. While heart function improved in both groups, the group treated with both cell types showed significant improvements with a greater reduction in cardiac fibrosis and increased the reparative activity of macrophages.

## Introduction

To date, inducing cardiac regeneration using stem cells has remained tremendously challenging. Bone marrow-derived mesenchymal stem cells (BMMSCs) are regarded as an attractive option for cardiac regeneration therapy^1^. BMMSCs are relatively easy to isolate and can differentiate into mesenchymal lineage cell types, such as osteocytes, chondrocytes, adipocytes, and myocytes^2^. Additionally, MSCs have the immunoprivileged capacity to avoid rejection, suppress inflammation in the lesion, and modulate immune cell phenotypes^3^. Despite the safety and feasibility of BMMSCs, concerns over their use remain due to equivocal clinical outcomes^4^. Various modifications have been proposed to improve the therapeutic efficacy of BMMSCs in cardiovascular disorders, such as priming with growth factors, cardiogenic cocktails, or apicidin or hypoxia-enhanced functional restoration via angiogenesis and cardiac differentiation^5–7^.

Macrophages are highly dynamic immune cells with diverse functions and are widely involved in the pathogenesis of cardiovascular diseases, including myocardial infarction (MI) and atherosclerosis. After MI, different leukocyte populations dynamically infiltrate the heart^8^. Importantly, anti-inflammatory macrophages are essential for inducing infarct healing, because depletion of cardiac macrophages drastically impairs healing and worsens the disease outcome^9^. Moreover, infarct healing and repair by BMMSCs is mediated by macrophages^10^. We previously reported that macrophages were shifted toward the anti-inflammatory phenotype by coculture with BMMSCs^11^.

Given the important contribution of anti-inflammatory macrophages to infarct healing, we hypothesized that macrophages cocultured with BMMSCs would be safe and effective adjuvant cells for cell therapy. To prove this concept, we prepared a mixture of BMMSCs and anti-inflammatory macrophages isolated and differentiated from bone marrow (BMDMs) and investigated their therapeutic efficacy in a rat MI model.

## Materials and Methods

### Cytokines and reagent

Recombinant rat interleukin-4 (IL-4), IL-13, and interferon-γ (IFN-γ) were purchased from Life Technologies (Grand Island, NY, USA). Lipopolysaccharide (LPS) was purchased from Sigma-Aldrich (St. Louis, MO, USA).

### Cell culture

Rat BMMSCs (rBMMSCs) were cultured in DMEM with 10% FBS (Gibco) and 1% penicillin/streptomycin (Gibco, Grand Island, NY, USA). The cells were grown at 37 °C in a humidified atmosphere with 95% air and 5% CO_2_, and the medium was changed every 3 days. On reaching confluence, the cells were resuspended with 0.25% trypsin-EDTA (Gibco) and reseeded at a 1:2 split ratio. The rBMMSCs were used within three passages. Human BMMSCs (hBMMSCs) immortalized by the introduction of telomerase were kindly provided by Professor Yeon-Soo Kim (Inje University, Inje, South Korea). Rat bone marrow cells or THP-1 cells were cultured in RPMI-1640 medium (Gibco) supplemented with macrophage differentiation medium (30% L929 cell-conditioned medium, 20% FBS, and 50% RPMI-1640). L929 cell-conditioned medium was prepared by growing L929 cells in RPMI-1640 medium containing 10% FBS for 10 days. The medium was harvested and passed through a 0.22-μm filter. The macrophages were treated with LPS (100 ng/mL)/IFN-γ (30 ng/mL) or IL-4 (20 ng/mL)/IL-13 (20 ng/mL) to induce polarization.

### Coculture of rBMDMs and rBMCs

For coculture, differentiated rBMDMs were seeded into a six-well plate. The next day, rBMMSCs were placed into 0.4-mm-pore size Corning Transwell culture plate inserts (Sigma-Aldrich).

### Real-time PCR

To compare mRNA expression levels, cells were harvested and homogenized in TRIzol solution (Invitrogen, Carlsbad, CA, USA) according to the manufacturer’s instructions. cDNA was generated using the TaqMan MicroRNA Reverse Transcription kit (Applied Biosystems, Foster Ckty, CA, USA), and real-time PCR was performed using the QuantiTect SYBR Green PCR Kit (Qiagen, Hilden, Germany) and Corbett Research Rotor-Gene RG-3000 Real Time PCR System. The sequence-specific human primers were purchased and rat primers were synthesized (Bioneer, Daejeon, Korea). The rat primers are described in Table 1.

**Table 1:** Primer sequences for the real-time PCR analysis

Gene	Forward primer	Reverse primer
Rat iNOS	TCACCTTCGAGGGCAGCCGA	CAGACGCCATGGTGCAGGGG
Rat Arg1	ATTCACCCCGGCTACGGGCA	AGGAGCAGCGTTGGCCTGGT
Rat IL-1β	ACCTGTCCTGTGTGATGAAAG	CTCCACTTTGGTCTTGACTTCT
Rat TNF-α	GCAGATGGGCTGTACCTTATC	GAAATGGCAAATCGGCTGAC
Rat GAPDH	GGCCAAGGTCATCCATGA	TCAGTGTAGCCCAGGATG
Rat CD206	GACGGACGAGGAGTTCATTATAC	GTTGGAGAGATAGGCACAGAAG
Rat IL-10	AGTGGAGCAGGTGAAGAATG	GAGTGTCACGTAGGCTTCTA

### Western blotting

Whole cell lysates were harvested using lysis buffer (20 mM Tris-HCl pH 7.4, 0.1 mM EDTA, 150 mM NaCl, 1 mM phenylmethylsulfonyl fluoride, and 1 mg/mL of leupeptin) on a rotation wheel for 1 h at 4 °C. After centrifugation at 10,000×*g* for 10 min, the supernatant was prepared as a protein extract. Equal concentrations of proteins were fractionated by electrophoresis on 8 to 12% acrylamide gels and transferred onto a polyvinylidene fluoride membrane (Merck Millipore, Darmstadt, Germany). The membranes were blotted with antibodies against iNOS, Arg1, IRF5 (Cell Signaling Technology, Danvers, MA, USA) and GAPDH (Santa Cruz Biotech, Dallas, TX, USA), followed by secondary antibodies. Protein expression was detected using an Image Reader (LAS-3000 Imaging System, Fuji Photo Film, Tokyo, Japan).

### Cell transplantation in a rat myocardial infarction model

Male inbred SD mice (approximately 7–8 weeks of age) were purchased from SAMTAKO Inc. (Korea). All experiments were performed after receiving approval from our local ethics committee at Chonnam National University Medical School (CNU IACUC-H-2014–22). The mice were anesthetized with an intramuscular injection of ketamine (50 mg/kg) and xylazine (5 mg/kg), intubated, and mechanically ventilated. The proximal left anterior descending coronary artery was ligated. For transplantation into the hearts, rBMMSCs (5 × 10^5^ diluted in 200 µL of PBS) and a mixture of rBMDMs and rBMMSCs (5 × 10^5^ diluted in 200 µL of PBS) were prepared. PBS (*n* = 11), rBMCs (*n* = 8), or MIX (rBMDMs + rBMMSCs, *n* = 8) was injected into the peri-infarct area of the left ventricular myocardium. Finally, the heart was repositioned in the chest, and the chest was closed. The animals remained in a supervised setting until they fully regained consciousness.

### Cardiac function measurement

At 2 weeks, cardiac function was assessed by transthoracic echocardiography (15-MHz linear array transducer system; iE33 system, Philips Medical Systems; Amsterdam, the Netherlands).

### Histological analyses and immunohistochemistry

For the immunohistochemical analysis, the heart was harvested, embedded in Tissue Tek O.C.T. compound (Leica, Germany), and frozen in liquid nitrogen. Frozen tissues were cut at a thickness of 10 μm and mounted onto glass slides for staining. Cardiac fibrosis was measured by Masson’s trichrome staining, and fibrotic changes were evaluated by measuring blue-stained fibrotic deposits using an Eclipse-80i microscope and the NIS-Elements software (Nikon, Tokyo, Japan). The percentage of ventricular fibrosis was calculated as the blue-stained area divided by the total ventricular area. For the immunohistochemical analysis, the slides were fixed with acetone for 10 min, permeabilized with 0.1% Triton X-100 for 10 min, and treated with 3% hydrogen peroxide in PBS for 10 min at room temperature to block endogenous peroxidase activity. After blocking nonspecific binding with 5% normal goat serum or 5% normal horse serum (Vector Laboratories, Burlingame, CA, USA) at room temperature for 1 h, the excess serum was removed. The slides were incubated with primary antibodies against CD68 (BMA Biomedicals, Switzerland, 1:100), CD206 (Abcam, Cambridge, MA, USA, 1:100), and von Willebrand Factor (vWF; Sigma-Aldrich) for 24 h at 4 °C. The sections were washed with PBS three times and then incubated for 1 h with Alexa-Fluor 488 or 594 secondary antibodies. After washing, the slides were coverslipped with mounting medium (VectaMount mounting medium, Vector Laboratories). Images were obtained using the NIS-Elements Advanced Research Program (Nikon, Japan).

### siRNA transfection

RNA interference was performed according to the manufacturer’s protocols. Briefly, a siRNA-control (si-Con) and siRNA specific for IRF5 (si-IRF5) were purchased from Bioneer (Daejeon, South Korea). hBMCs were transfected with the siRNAs using the Lipofectamine RNAiMAX transfection reagent (Invitrogen). The cells were allowed to grow for another 48 h before being collected for the IRF5 knockdown experiments.

### Statistical analysis

Each experiment was performed at least three times. The data are presented as the means ± SDs. Differences were analyzed by Student’s *t*-test or ANOVA and were considered significant when the *P*-value was <0.05.

## Results

**Fig. 1:**
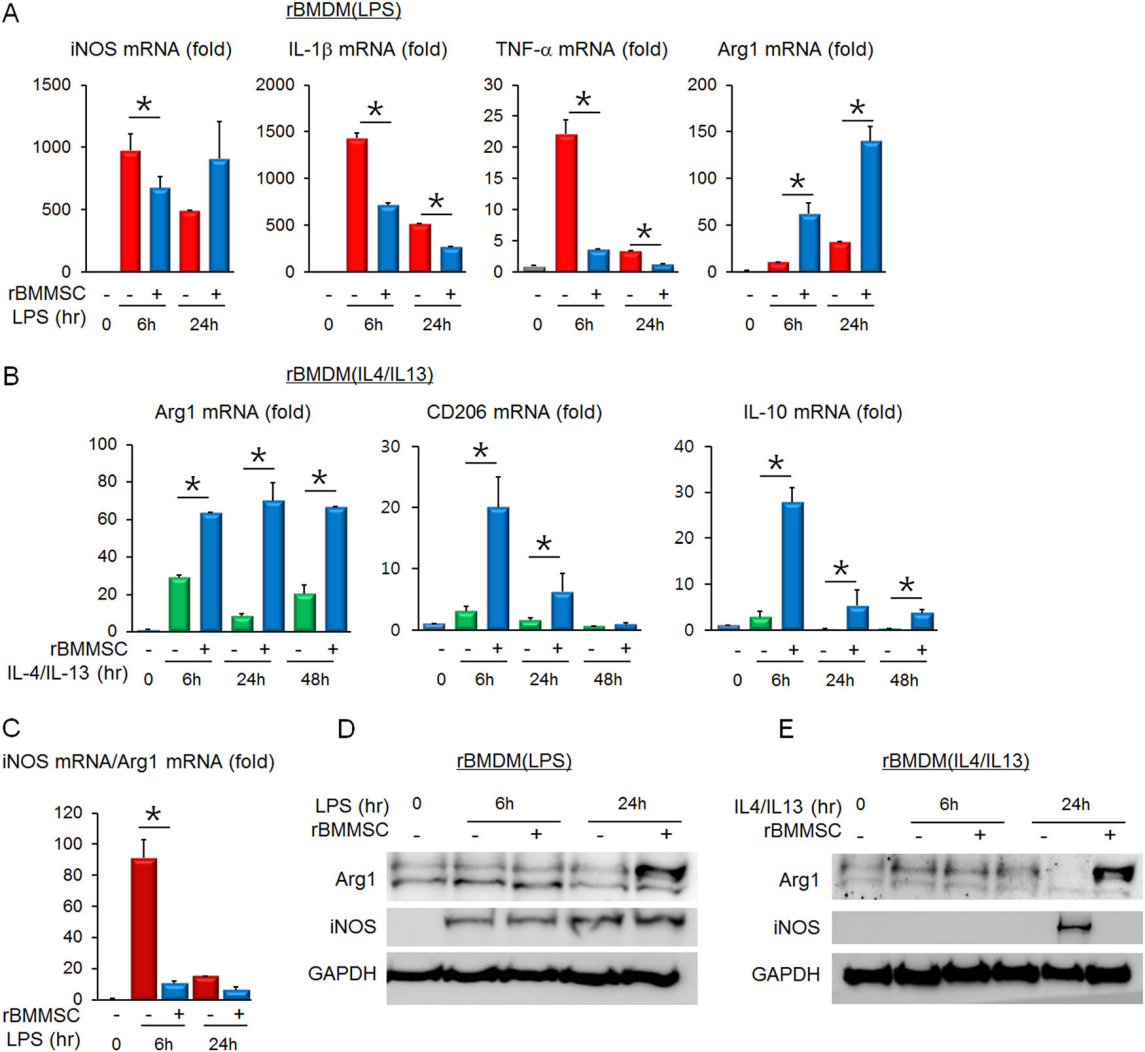
Expression of inflammation-related mediators in rBMDMs is altered by coculture with rBMMSCs. **a** The mRNA levels of factors such as iNOS, IL-1β, TNF-α, and Arg1 were assessed in LPS-stimulated BMDMs with or without rBMMSC coculture. **b** The mRNA levels of factors such as Arg1, CD206, and IL-10 were assessed in IL-4/IL-13-treated BMDMs with or without rBMMSC coculture. **c** The iNOS mRNA to Arg1 mRNA ratio was calculated to show the skewed macrophage polarization toward the anti-inflammatory phenotype. The Arg1 and iNOS protein expression patterns in LPS-stimulated rBMDMs (**d**) or IL-4/IL-13-treated rBMDMs (**e**) with or without rBMMSC coculture. rBMDMs Rat bone marrow-derived macrophages; rBMMSCs Rat bone marrow-derived mesenchymal stem cells

**Fig. 2:**
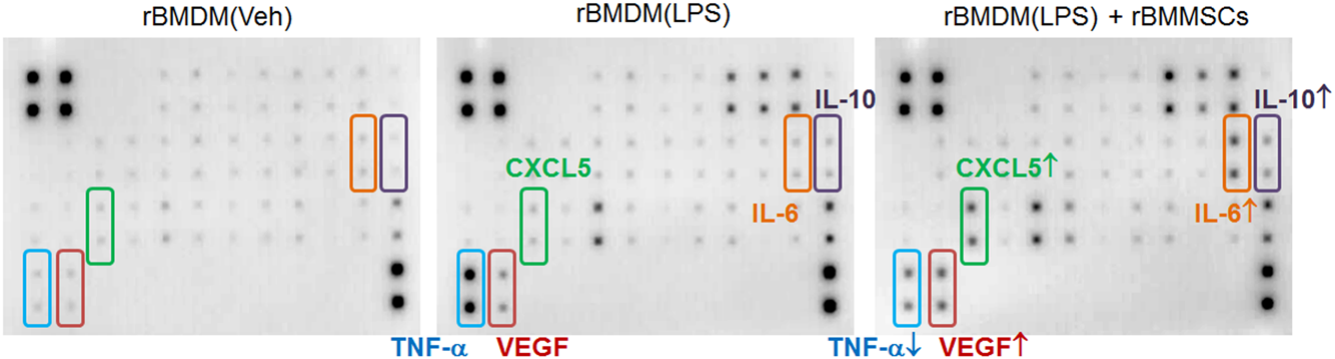
Antibody array showing that the soluble mediator profiles from BMDMs are altered by coculture with BMMSCs. rBMDM (Veh) Vehicle-treated rat BMDMs; rBMDM (LPS) LPS-stimulated rat BMDMs; rBMDM (LPS) + rBMMSCs, LPS stimulation of rBMDMs followed by coculture with rBMMSCs

rBMMSCs and rBMDMs were isolated and differentiated for coculture. First, the mRNA expression levels of inflammatory mediators were measured in LPS-stimulated rBMDMs. Coculture with rBMMSCs reduced the mRNA induction of proinflammatory genes, including iNOS, TNF-α, and IL-1β, whereas the anti-inflammatory Arg1 mRNA was upregulated in the LPS-stimulated rBMDMs (Fig. 1a). Then, the mRNA levels of anti-inflammatory mediators were assessed. Arg1, CD206 and IL-10 were highly induced by culture of the IL-4/IL-13-treated rBMDMs with the rBMMSCs (Fig. 1b). Because proinflammatory iNOS and anti-inflammatory Arg1 are the representative markers in rodent monocytes and macrophages, the iNOS to Arg1 ratio was expressed as an indicator of the rBMDM inflammatory status. As shown in Fig. 1c, coculture with rBMMSCs drastically suppressed the inflammatory phenotype of the rBMDMs. Then, the iNOS and Arg1 protein levels were determined by Western blotting. Arg1 was highly increased in LPS-stimulated rBMDMs cocultured with rBMMSCs. However, the iNOS levels were not significantly different (Fig. 1d). In the IL-4/IL-13-treated rBMDMs, Arg1 was markedly upregulated, whereas iNOS induction was blocked by BMMSC coculture (Fig. 1e). The overall expression pattern of inflammatory mediators showed high induction of anti-inflammatory Arg1, as well as low expression of proinflammatory mediators in rBMDMs cocultured with rBMMSCs. In addition to iNOS and Arg1, soluble factors released from the rBMDMs were analyzed using an antibody array. The rBMDMs were treated with LPS with or without coculture with rBMMSCs. After 24 h, the culture media were collected for the analysis. In the antibody array, TNF-α was reduced and VEGF, CXCL5, IL-6, and IL-10 were increased in BMDMs cocultured with BMMSCs compared with the levels in BMDMs cultured alone (Fig. 2).

**Fig. 3:**
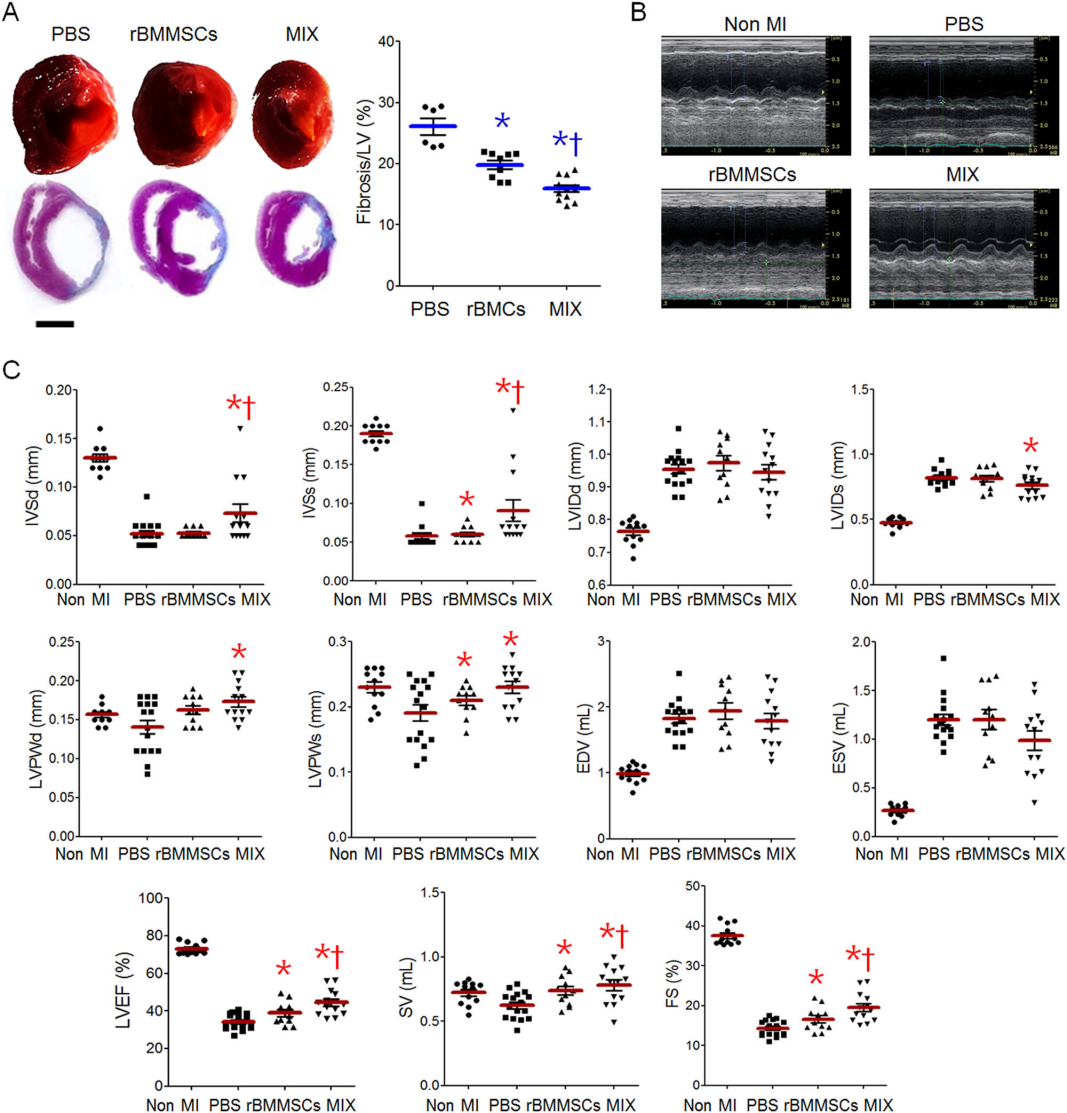
Therapeutic efficacies of BMCs and MIX in a rat myocardial infarction (MI) model. **a** Representative gross images (upper) and trichrome-stained heart tissues (lower) from the MI model rats injected with PBS, rBMMSCs, or MIX. Cardiac fibrosis was quantified (right). **b** Representative echocardiograms of the non-MI, PBS, rBMMSC, and MIX groups. **c** The cardiac function indexes were measured by echocardiography

These results showed that the phenotype of the rBMDMs clearly shifted to anti-inflammatory after coculture with rBMMSCs. Thus, we studied whether the anti-inflammatory rBMDMs would support the therapeutic efficacy of rBMMSCs in a rat MI model. To this end, we injected PBS, rBMMSCs, or a mixture of rBMMSCs and rBMDMs (MIX) into the infarcted myocardium and assessed cardiac fibrosis and function after 2 weeks. The extents of cardiac fibrosis determined by Masson’s trichrome staining were 26.1 ± 3.3%, 19.8 ± 2.1%, and 15.9 ± 1.9% in the PBS, rBMMSC, and MIX groups, respectively (Fig. 3a). Cardiac function was assessed by echocardiography (Fig. 3b). The MI characterization exhibited better improvement in the MIX group than in the rBMMSC group, although systolic function indexes, such as LVEF, SV and LVFS, also recovered in the rBMMSC group (Fig. 3c).

**Fig. 4:**
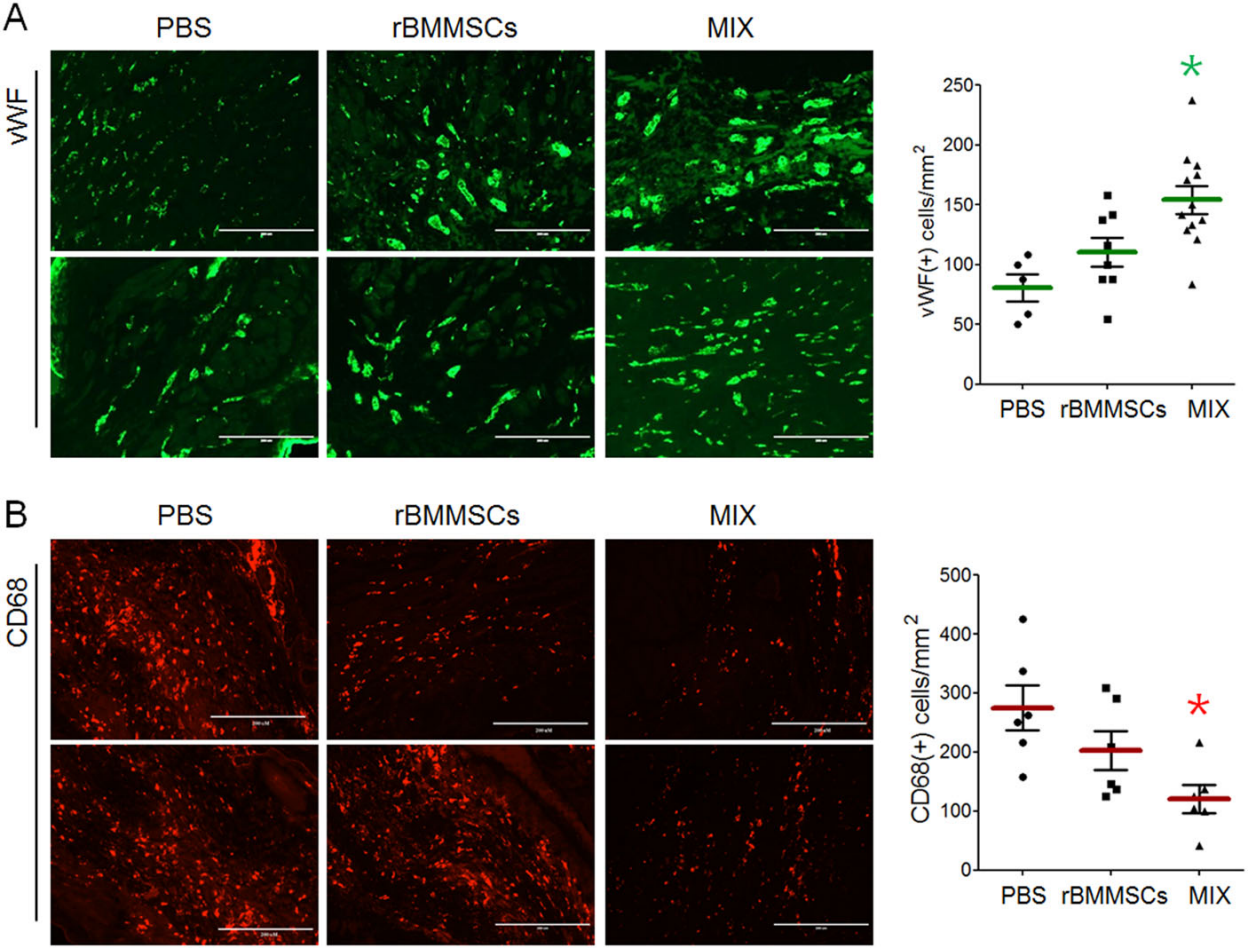
Angiogenesis and macrophage infiltration in the infarcted myocardium. **a** The infarcted myocardium was immunostained for vWF to analyze angiogenesis in the PBS, rBMMSC, and MIX groups, and vWF-positive cells were counted for quantification. **b** Immunostaining with CD68 showed macrophage infiltration, and the number of CD68-positive cells was quantified

Furthermore, angiogenesis and CD68-positive macrophage infiltration were determined by immunohistochemical staining. The increase in the number of vWF-positive cells was larger in the MIX group than in the rBMMSC group (Fig. 4a). Infiltration of CD68-positive cells was reduced in the rBMMSC group and was much lower in the MIX group than in the PBS group (Fig. 4b).

**Fig. 5:**
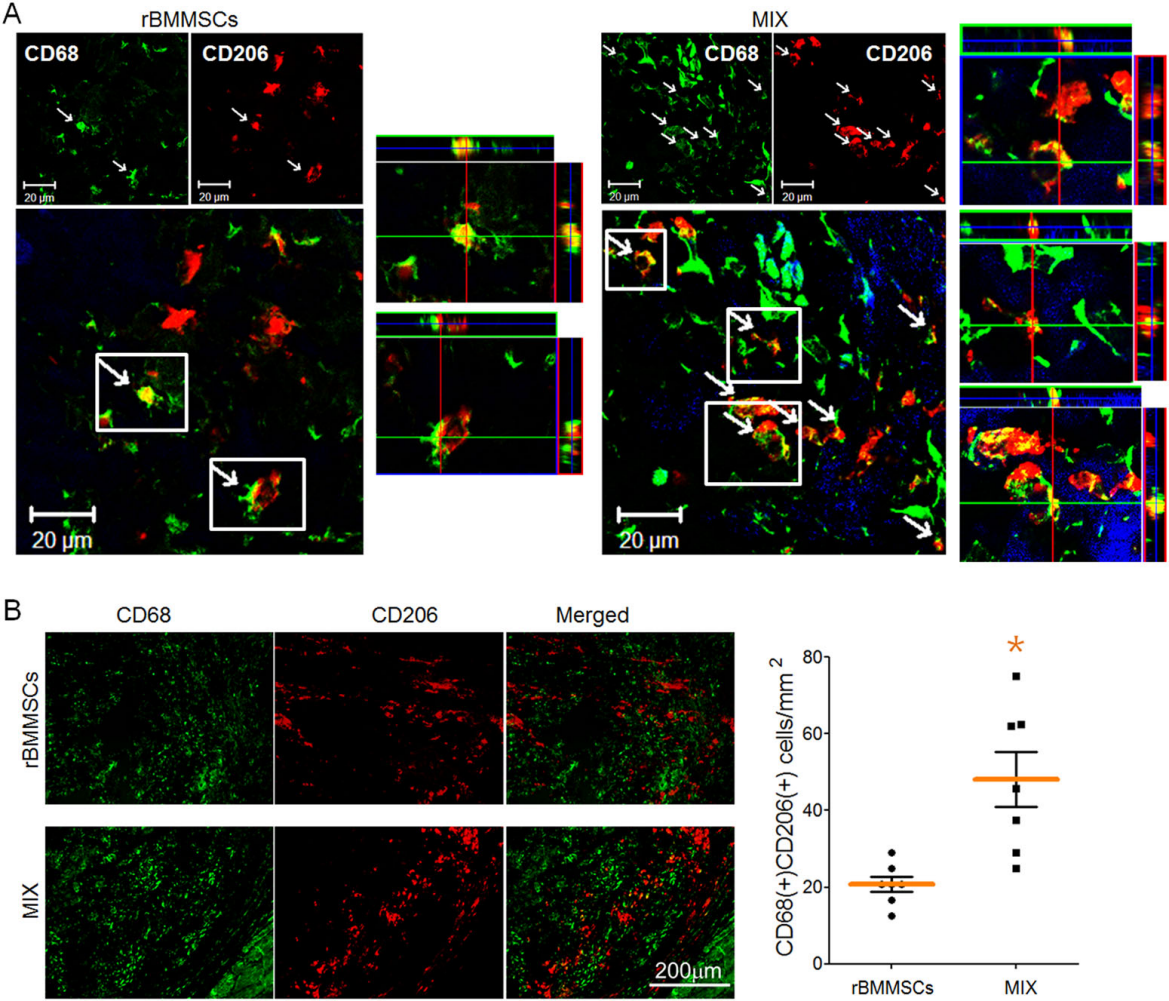
Representative images showing that anti-inflammatory macrophages are more abundant in the MIX group than in the BMMSC group. **a** Anti-inflammatory macrophages were detected by double staining with the macrophage marker CD68 and the anti-inflammatory marker CD206 in confocal images. **b** The number of anti-inflammatory macrophages identified by double-positive staining for CD68 and CD206 was higher in the MIX group than in the rBMMSC group

Next, we determined the phenotype of macrophages in the infarcted heart by double immunohistochemical staining for CD68 (a macrophage marker) and CD206 (an anti-inflammatory macrophage marker). In the confocal images, double-positive cells were more frequently observed in the MIX group than in the rBMMSC group (Fig. 5a). Anti-inflammatory macrophages were quantified by counting CD68-positive and CD206-positive cells; the number of these cells was much higher in the MIX group than in the rBMMSC group (Fig. 5b).

**Fig. 6:**
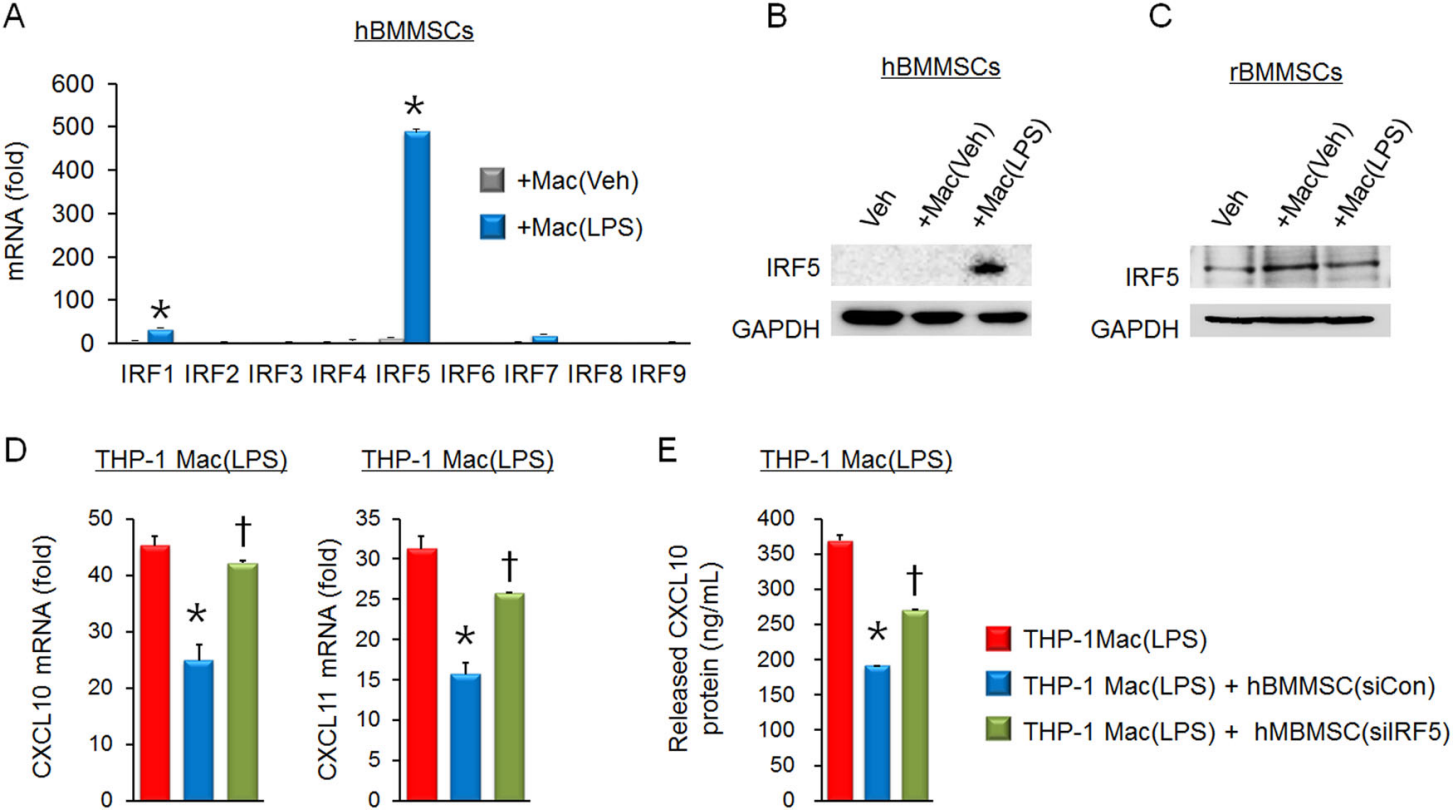
IRF5 is essential in BMCs and exerts an anti-inflammatory effect on LPS-stimulated macrophages. **a** mRNA expression patterns of IRF family members were examined in hBMMSCs after coculture with THP-1-derived macrophages stimulated with vehicle or LPS. **b** IRF5 protein expression was upregulated in hBMMSCs cocultured with LPS-stimulated macrophages. **c** IRF5 protein expression also increased in rBMMSCs cocultured with rBMDMs. **d** LPS-induced upregulation of the proinflammatory markers CXCL10 and CXCL11 was apparently inhibited by coculture with hBMMSCs transfected with the siRNA control. Conversely, coculture with hBMMSCs transfected with the IRF5 siRNA failed to attenuate the upregulation of CXCL10 and CXCL11. **e** CXCL10 protein expression was measured in media from THP-1-differentiated macrophages cocultured with hBMMSCs transfected with the siRNA control or IRF5 siRNA

Next, we analyzed the gene expression patterns in human BMCs to define the factor responsible for driving macrophages toward an anti-inflammatory phenotype and selected interferon regulatory factor 5 (IRF5) as a prominent candidate. Hereafter, human BMMSCs (hBMMSCs) and differentiated human THP-1 cells were used due to technical problems, such as transfection difficulty. In the hBMMSCs, the IRF mRNA expression patterns were analyzed. A huge increase in IRF5 was seen in hBMMSCs cocultured with LPS-stimulated macrophages (Fig. 6a). Additionally, IRF5 protein expression was induced by coculture with macrophages with both the hBMMSCs and rBMMSCs (Fig. 6b, c).

Next, to determine whether IRF5 was a critical factor in BMCs for exertion of anti-inflammatory effects on macrophages, IRF5 knockdown was induced in hBMMSCs by siRNA transfection. CXCL10 and CXCL11 were used as inflammatory markers of differentiated THP-1 macrophage cells. The IRF5-knockdown hBMMSCs failed to inhibit the mRNA induction of CXCL10 and CXCL11 (Fig. 6d). Moreover, LPS-induced CXCL10 protein expression was significantly inhibited by coculture with control siRNA-transfected hBMMSCs but was partially restored by coculture with IRF5 siRNA-transfected hBMMSCs (Fig. 6e).

## Discussion

The results of the present study suggest an adjuvant role for anti-inflammatory macrophages as efficient stem cell therapy for MI. Macrophages are key effectors of immune cells in response to tissue injury and are emerging as therapeutic targets for inflammation-related disorders, including MI and atherosclerosis^12–15^. The murine heart contains myeloid cells with approximately 7 to 8% noncardiomyocytes^16^, and inflammatory macrophages have been reported to contribute to the initiation and propagation of adverse cardiac remodeling during cardiovascular healing processes. The associations between macrophage subsets and functional repair have been validated in independent studies.

The main cells used for stem cell therapy for cardiac regeneration are MSCs, cardiac progenitor cells, and differentiated endothelial cells^17–19^. In clinical studies of ischemic heart disease, improvement was shown in the left ventricular ejection fraction in only 8 of 22 trials, and this analysis concluded that adult stem cell therapy was not ready for routine clinical application^20^. The field of cell therapy for cardiovascular disease has undergone extensive studies and clinical studies using BMMSCs, which exhibit safety profiles and moderate improvement of acute MI and heart failure. However, therapeutic efficacy should be studied in large randomized controlled trials with long-term follow-up. Multiple translational failures led researchers to return to the benches and develop distinguished approaches, such as combined transplantation of different stem cell types. An ongoing clinical study (CONCERT-HF) is evaluating the feasibility, safety, and effect of a combination of MSCs and c-kit(+) cardiac stem cells on heart failure (NCT02501811)^21^.

A great deal of supportive data are available concerning the functions of cardiac macrophages under physiological and pathological conditions. Although the diverse macrophage phenotypes have not been fully characterized, macrophages have been classified into several subsets to clarify their functions. Inflammatory monocytes and macrophages are predominant in the early phase after infarction. These cells produce inflammatory molecules, mediate proteolysis, and phagocytose cell debris in the infarcted heart tissue. In the later stages, alternatively activated macrophages are involved in the repair process and contribute to wound healing and angiogenesis through the production of anti-inflammatory cytokines and growth factors^22,23^.

The induction of anti-inflammatory cytokines is initiated by apoptotic cell ingestion in macrophages. Application of phosphatidylserine-presenting liposomes to mimic apoptotic cells shifted the macrophage phenotype to anti-inflammatory and resulted in the successful prevention of left ventricle remodeling and heart dilatation^24,25^. Interestingly, the depletion of macrophages in mice with MI increased mortality, left ventricular rupture and fibrosis^26,27^. Furthermore, macrophages were required for heart regeneration in stem cell therapy, including MSCs and cardiosphere-derived cells^10,28^, and transplant of these stem cells into the infarcted myocardium induced macrophage polarization toward the reparative phase.

As discussed above, we realized that anti-inflammatory macrophages were clearly involved in the cardiac repair process, and we designed the present study to recapitulate the reparative microenvironment with anti-inflammatory macrophages. Recently, MSCs have been well recognized as a modulator of the immune response that can attenuate the systemic inflammatory response mediated by macrophages^11,29^. Therefore, we harvested anti-inflammatory macrophages after coculture with BMMSCs and then transplanted the mixture of BMMSCs and cocultured macrophages into infarcted heart tissue. This protocol was designed to show that macrophages educated by BMMSCs could be utilized as a cellular adjuvant to limit pathological progression.

A cell therapy product has a concept similar to ours in terms of the cell composition and source. Ixmyelocel-T was developed by culturing a patient’s bone marrow in an automated closed-culture system over 11–13 days^30^. Further analysis showed that the ixmyelocel-T product was primarily composed of CD90(+) mesenchymal stromal cells and CD14(+) macrophages with high expression levels of anti-inflammatory markers, including IL-10, CD163 and CD206^31^. Moreover, ixmyelocel-T showed an enhanced cholesterol-efflux capacity and was suggested to be beneficial for atherosclerosis^32^. In clinical trials, IMPACT-DCM, ixCELL-DCM, and ixmyelocel-T were applied to treat dilated cardiomyopathy via a catheter-based transendocardial injection^33,34^. A clinically meaningful reduction of cardiac events was observed in patients with ischemic dilated cardiomyopathy but not in those with nonischemic dilated cardiomyopathy. Structural changes in the left ventricular cavity size were not noted in the ixmyelocel-T group at month 12.

IRF5 is a definite factor for commitment to the inflammatory macrophage lineage, is reported to be expressed on neutrophils^35,36^, and plays an important role in controlling the innate immune response^37^. Silencing IRF5 improves infarct healing and attenuates heart failure via accelerated resolution of inflammation in the mouse MI model^38^. However, the function of IRF5 in BMMSCs has not been reported elsewhere. Of the IRF family members, only IRF5 was highly induced in BMMSCs following coculture with macrophages. Moreover, knockdown of IRF5 in BMMSCs blunted their anti-inflammatory activity against LPS-stimulated macrophages. The mechanism by which BMMSCs regulate inflammatory pathways is incompletely understood. IRF5 may be a supportive factor for BMMSCs in terms of immune modulatory activity.

From a clinical perspective, the most prominent advantage of using BMMSCs and BMDMs is that these two cell types originate from the same donor’s bone marrow. In other words, bone marrow aspirated from a patient is sufficient to produce both BMMSCs and BMDMs for improved cell therapy. This protocol does not require additional pretreatments with reagents, genetic modifications, or differentiation induction for functional enhancement.

Our study had several limitations. First, we experienced technical difficulties with siRNA transfection in rBMMSCs. Second, we were not successful in distinguishing the injected BMDMs from resident macrophages in the infarcted myocardium.

**Fig. 7:**
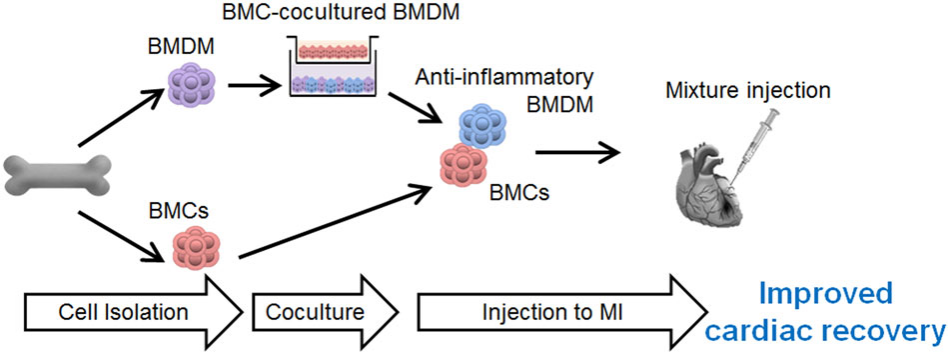
**Schematic representation of the contribution of BMMSC-primed BMDMs to the overall improvement in BMC-induced cardiac repair**

Overall, this study showed that BMMSCs and BMDMs harvested from the same donor could be applied for cell therapy following by a coculture procedure. Cotransplantation of BMMSCs and educated BMDMs successfully induced significant functional and structural improvement in the MI rat model. Taken together, these data suggest that induction of IRF5 in BMMSCs is essential for suppressing anti-inflammatory phenotypic changes of macrophages. We suggest that BMDMs may be a feasible and effective adjuvant candidate to enhance stem cell therapy (Fig. 7).
